# Plk1 and CK2 Act in Concert to Regulate Rad51 during DNA Double Strand Break Repair

**DOI:** 10.1016/j.molcel.2011.12.028

**Published:** 2012-02-10

**Authors:** Keiko Yata, Janette Lloyd, Sarah Maslen, Jean-Yves Bleuyard, Mark Skehel, Stephen J. Smerdon, Fumiko Esashi

**Affiliations:** 1 Sir William Dunn School of Pathology, University of Oxford, South Parks Road, Oxford OX1 3RE, UK; 2 Division of Molecular Structure, MRC National Institute for Medical Research, The Ridgeway NW7 1AA, UK; 3 Cancer Research UK, London Research Institute, Clare Hall Laboratories, South Mimms, Herts EN6 3LD, UK

## Abstract

Homologous recombination (HR) plays an important role in the maintenance of genome integrity. HR repairs broken DNA during S and G2 phases of the cell cycle but its regulatory mechanisms remain elusive. Here, we report that Polo-like kinase 1 (Plk1), which is vital for cell proliferation and is frequently upregulated in cancer cells, phosphorylates the essential Rad51 recombinase at serine 14 (S14) during the cell cycle and in response to DNA damage. Strikingly, S14 phosphorylation licenses subsequent Rad51 phosphorylation at threonine 13 (T13) by casein kinase 2 (CK2), which in turn triggers direct binding to the Nijmegen breakage syndrome gene product, Nbs1. This mechanism facilitates Rad51 recruitment to damage sites, thus enhancing cellular resistance to genotoxic stresses. Our results uncover a role of Plk1 in linking DNA damage recognition with HR repair and suggest a molecular mechanism for cancer development associated with elevated activity of Plk1.

## Graphical abstract

**Graphical Abstract:**

**Figure:**
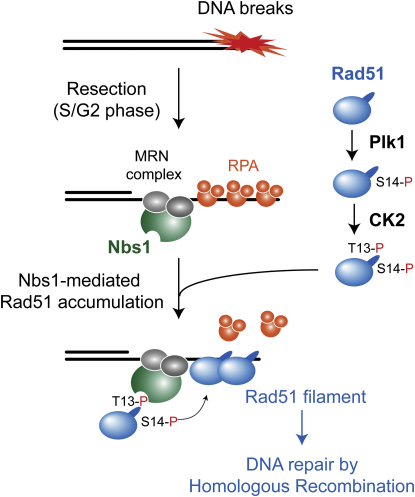

**Highlights:**

► Plk1 phosphorylates Rad51 recombinase at S14 in a highly specific manner ► Rad51 phosphorylation at S14 triggers subsequent phosphorylation at T13 by CK2 ► T13-phosphorylated Rad51 binds to the Nijmegen breakage syndrome gene product, Nbs1 ► Rad51 binding to Nbs1 is important for cellular resistance to genotoxic stresses

## Introduction

Precise repair of DNA double-strand breaks (DSBs) that are caused during DNA replication and by exogenous stresses such as ionizing radiation (IR) is critical for the maintenance of genome integrity. Accurate regulation of homologous recombination (HR), which repairs DSBs using the replicated sister chromatid as a repair template, is important during S and G2 phases of the cell cycle. Downregulation of HR results in chromosomal rearrangements due to the engagement of alternative error-prone DSB repair mechanisms such as nonhomologous end-joining (NHEJ), whereas hyperrecombination also causes various genome instability phenotypes including loss of heterozygosity, gene amplification, and gene deletion (Stankiewicz and Lupski, 2002; van Gent et al., 2001).

Nijmegen breakage syndrome (NBS) is an autosomal recessive chromosomal instability syndrome, and cells defective in the *NBS1* gene exhibit increased sensitivity to IR (Digweed et al., 1999; Varon et al., 1998). Nbs1, together with its binding partners Mre11 and Rad50, is efficiently recruited to damaged chromatin via Mdc1 (mediator of DNA damage checkpoint 1) and also directly recruited to single-stranded DNA (ssDNA) (Bekker-Jensen et al., 2006; Chapman and Jackson, 2008). These events are critical for checkpoint activation and signal amplification. The recruited Mre11-Rad50-Nbs1 (MRN) complex also assists in the repair of DSBs; the complex holds two DSB ends together to facilitate nonhomologous end-joining (Rass et al., 2009; Xie et al., 2009) or, when cells are in S or G2, promotes DSB resection to initiate HR (Stracker and Petrini, 2011; Tauchi et al., 2002). The ssDNA generated from resection of double-stranded DNA (dsDNA) is subsequently bound by the single-strand binding protein RPA (replication protein A), which is then replaced by the Rad51 recombinase, which catalyzes homologous pairing and strand transfer during HR (West, 2003; Wyman and Kanaar, 2004). Recruitment and activity of Rad51 are stimulated by additional factors, most critically by the tumor suppressor, breast cancer 2 (BRCA2) (Venkitaraman, 2002; West, 2003). *BRCA2* was originally identified through germ-line mutations that predispose individuals to the development of breast and ovarian cancers (Lancaster et al., 1996). *BRCA2*-defective cell lines exhibit spontaneous gross chromosomal instability, HR-defective phenotypes, and elevated sensitivity to IR during S and G2 (Connor et al., 1997; Tutt et al., 2003). Studies using purified full-length BRCA2 suggest that BRCA2 stimulates Rad51 loading onto RPA-coated ssDNA (Jensen et al., 2010; Liu et al., 2010; Thorslund et al., 2010). Nonetheless, Rad51 associates with chromatin during DNA replication in *BRCA2*-defective cells (Tarsounas et al., 2003), and elevated expression of Rad51, which is often found in radioresistant cancer cells, bypasses the BRCA2 dependency of HR repair (Brown and Holt, 2009; Lee et al., 2009). A recent epistasis study using the DT40 system also supports the notion that Rad51 performs HR independently of BRCA2 (Qing et al., 2011). Together, these observations suggest that Rad51 recruitment to damage sites can also be mediated through BRCA2-independent mechanisms.

HR processes are temporally controlled by the central cell-cycle regulators, cyclin-dependent kinases (CDKs) (Esashi et al., 2005; Huertas et al., 2008; Ira et al., 2004; Jazayeri et al., 2006; Yun and Hiom, 2009) but a complete picture of cell cycle-dependent HR regulation remains elusive. In addition to CDKs, Polo-like kinase 1 (Plk1) is increasingly recognized as an essential cell-cycle regulator. Although roles of Plk1 in mitosis are well documented, roles in interphase are also suggested by its nuclear accumulation in S and G2 and by its involvement in DNA replication and DNA damage responses (Takaki et al., 2008). Plk1 is structurally characterized by the polo-box domain (PBD) at the carboxyl terminus, which mediates its binding to phosphorylated proteins at specific intracellular locations. Subsequently, Plk1 phosphorylates binding partners and/or other local proteins and, hence, coordinates phosphorylation in a spatiotemporal manner (Barr et al., 2004; Elia et al., 2003). Importantly, proteomic and bioinformatic screens for PBD-binding proteins identified proteins involved in the damage response and HR repair, including Mdc1 and BRCA2 (Lowery et al., 2007; Lowery et al., 2005). It is unknown, however, whether and how Plk1 that binds to DNA damage responsive proteins may regulate DNA repair.

Here, we report a role of Plk1 in the DNA damage response wherein it directly phosphorylates Rad51 at residue S14 in its N-terminal regulatory domain. Significantly, S14 phosphorylation stimulates subsequent phosphorylation of Rad51 at T13 by casein kinase 2 (CK2), creating a phospho-binding motif for the Nbs1 Forkhead-associated (FHA) domain. Cells expressing Rad51 variants that retain binding to Nbs1, but not those with impaired Nbs1 binding, were rendered resistant to genotoxic stresses independently of BRCA2 function, whereas dynamic interaction between Nbs1 and Rad51 was important for precise HR repair of DSB. These findings demonstrate a mechanism for genome integrity control by Plk1-dependent phosphorylation of Rad51.

## Results

### Plk1 Phosphorylates the Rad51 Recombinase

**Figure 1:**
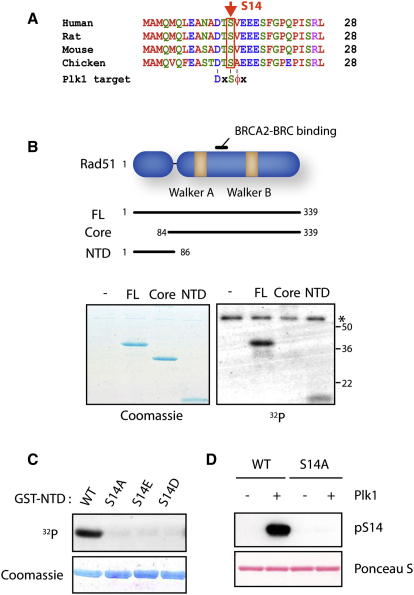
Plk1 Phosphorylates Rad51 at S14 In Vitro (A) Sequence alignment of the Rad51 N-terminal region. A canonical Plk1 target sequence and the Plk1 target residue are indicated. (B) Top, schematic illustration of full-length Rad51 (FL), ATPase core domain (Core), and amino-terminal domain (NTD). The Rad51 region that interacts with the BRCA2 BRC motif is also indicated. Bottom, kinase reactions were performed in the presence of γ^32^P-ATP, and ^32^P-labeled products were detected by autoradiography. The asterisk indicates autophosphorylation of Plk1. (C) Recombinant Rad51 NTD variants were phosphorylated with Plk1 in the presence of γ^32^P-ATP as above. (D) Recombinant Rad51 NTD variants were phosphorylated with Plk1, and S14 phosphorylation was detected by pS14 antibody.

We noticed that human Rad51 contains a canonical Plk1 target sequence ([D/E/N/Y]-x-pS/pT-[Φ/F]-[Φ/x]: x, any amino acid; Φ, a hydrophobic amino acid) (Alexander et al., 2011) near its N-terminus, where serine residue 14 is predicted to be phosphorylated by Plk1 (Figure 1A). Consistently, when a Plk1 kinase reaction was performed in the presence of γ^32^P-ATP, efficient incorporation of ^32^P into full-length Rad51 and the N-terminal Rad51 domain (residues 1–86, hereafter designated as NTD) but not the ATPase core domain (residues 84–339), was detected (Figure 1B). No phosphorylation was found with NTD fragments that harbor S14 mutation to alanine (S14A), aspartate (S14D), or glutamate (S14E), indicating that Plk1 phosphorylates S14 in vitro (Figure 1C). S14 phosphorylation of in vitro phosphorylated Rad51 was also confirmed using mass spectrometry (Figure S1B available online) and by phospho-S14 antibody (pS14; Figure 1D).

**Figure 2:**
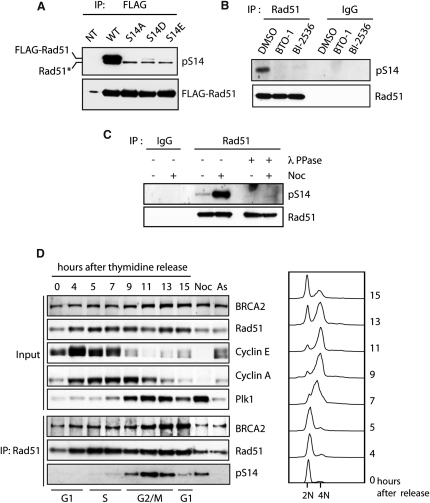
Rad51 Is Phosphorylated at S14 in a Plk1-Dependent Manner In Vivo (A) FLAG-tagged Rad51 variants purified from HEK293T cells were blotted with pS14 antibody or FLAG antibody. NT, nontransfected cells. The asterisk indicates endogenous Rad51 copurified with FLAG-Rad51. (B) HeLa cells were treated with DMSO or Plk1 inhibitors (BTO-1 or BI-2536), and immunoprecipitated Rad51 was analyzed using pS14 antibody. (C) Rad51 from HeLa cells treated with DMSO or nocodazole (Noc) was analyzed using pS14 antibody as above. No signal was detected after λ phosphatase (λ PPase) treatment, showing the phospho-specificity of the antibody. (D) HeLa cells were synchronized by double thymidine-block release, and Rad51 phosphorylation was analyzed. Cell-cycle progression was monitored by cell-cycle markers cyclin E (for G1/S), cyclin A (for S/G2), and Plk1 (for S/G2/M) and by FACS. As, asynchronous cells.

Next, we examined whether Rad51 is also phosphorylated in vivo at S14. First, N-terminally FLAG-tagged Rad51 and its S14 variants were transiently expressed in HEK293T cells, and FLAG-purified Rad51 fusions were blotted with the phospho-specific antibody. The pS14 antibody detected signals from wild-type (WT) Rad51 but not from the S14 variants (Figure 2A), demonstrating that exogenously expressed WT Rad51 was competently phosphorylated at S14. Importantly, endogenous Rad51 in HeLa cells was also detected with the pS14 antibody but this phosphorylation was substantially reduced when cells had been treated with Plk1 inhibitors (Figure 2B). Conversely, an increased signal was detected with this antibody when cells were arrested in mitosis with nocodazole, suggesting cell-cycle dependency of the S14 phosphorylation (Figure 2C). Indeed, detailed analyses of synchronized HeLa cells showed that the S14 phosphorylation gradually increased from S phase and peaked in early mitosis, which correlates with the Plk1 expression profile (Figure 2D). These observations show that Rad51 is phosphorylated at S14 by Plk1 in vivo.

### Concerted Phosphorylation of Rad51 by Plk1 and CK2

**Figure 3:**
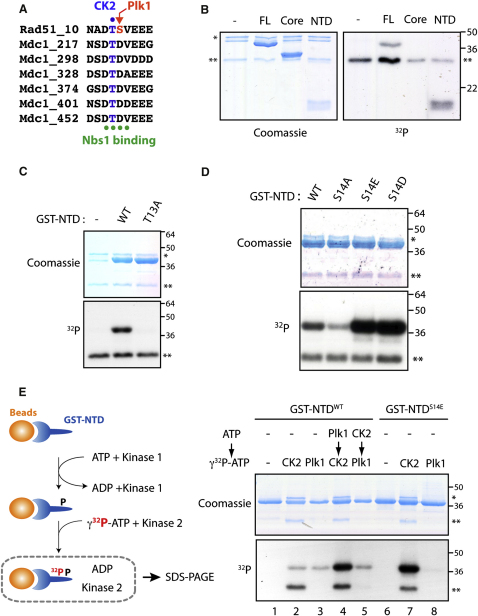
Sequential Phosphorylation of Rad51 by Plk1 and CK2 (A) Alignment of the Rad51 DTSV motif and Mdc1 SDTD motifs. The CK2 target residue, Plk1 target residue, and Mdc1 residues that interact with Nbs1 are highlighted with blue or red letters or with green dots, respectively. (B) Recombinant Rad51 as in Figure 1B was in vitro phosphorylated with CK2. (C) Recombinant Rad51 NTD and T13A variant were phosphorylated with CK2 as above. (D) Recombinant Rad51 NTD variants at the S14 site were phosphorylated with CK2 as above. (E) Left, schematic illustration for sequential phosphorylation analysis. Right, ^32^P-labeled products after sequential phosphorylation were detected by autoradiography. In panels (B), (C), (D) and (E), the asterisks indicate the CK2 α (^∗^) or β subunit (^∗∗^).

In an attempt to identify the molecular function of Plk1-mediated S14 phosphorylation, we carried out a short sequence BLAST analysis using the Plk1 phosphorylation motif in Rad51 and its surrounding sequence (hereafter designated as Rad51 DTSV motif). This analysis revealed a remarkable similarity to regions of Mdc1 containing SDTD motifs, a known target of CK2, where CK2 phosphorylation of the threonine between two acidic aspartate residues creates a binding site for Nbs1 (Figure 3A) (Chapman and Jackson, 2008; Lloyd et al., 2009; Melander et al., 2008; Spycher et al., 2008; Williams et al., 2009). Therefore, we examined the ability of CK2 to phosphorylate the threonine in the Rad51 DTSV motif. Notably, in vitro CK2 kinase assays revealed efficient phosphorylation of full-length Rad51 and the NTD fragment but not the ATPase core or a NTD fragment harboring an alanine substitution at threonine 13 (T13A) (Figures 3B and 3C), demonstrating that CK2 phosphorylates Rad51 at T13. Our mass spectrometric analysis of full-length Rad51 phosphorylated in vitro with CK2 also identified a single phosphorylation at T13 (Figure S1C).

Because CK2 is an acidophilic kinase, we next explored whether a negative charge at S14 modifies CK2-mediated phosphorylation at T13. Indeed, a Rad51 NTD fragment with S14 substituted by an acidic residue (D or E) was more susceptible to CK2 phosphorylation, whereas a NTD with S14A substitution was less efficiently phosphorylated (Figure 3D). These observations are consistent with the documented CK2 target preferences for highly acidic substrate motifs (Songyang et al., 1996) and the substantial deleterious effects on both *K*_m_ and *V*_max_ values for a CK2 peptide substrate with an alanine substitution at the pS/T +1 position (Sarno et al., 1997). We further evaluated whether Plk1 phosphorylation at S14, which renders this residue negatively charged, might modify the efficiency of CK2-mediated phosphorylation at T13. To this end, we first phosphorylated a GST fusion of Rad51 NTD immobilized on glutathione beads in the presence of nonradiolabeled ATP; after washing off the kinase and ATP, a second kinase reaction was carried out in the presence of γ^32^P-ATP (Figure 3E). Strikingly, prephosphorylation of Rad51 NTD with Plk1 substantially facilitated subsequent phosphorylation by CK2; in contrast, CK2-prephosphorylation of Rad51 did not alter the efficiency of Plk1-dependent phosphorylation (Figure 3E, compare lanes 2, 4, and 5). This result supports the view that Rad51 is sequentially modified by Plk1-dependent priming phosphorylation at S14, followed by CK2-mediated phosphorylation at T13.

### Dynamic Phosphorylation of Rad51 In Vivo

**Figure 4:**
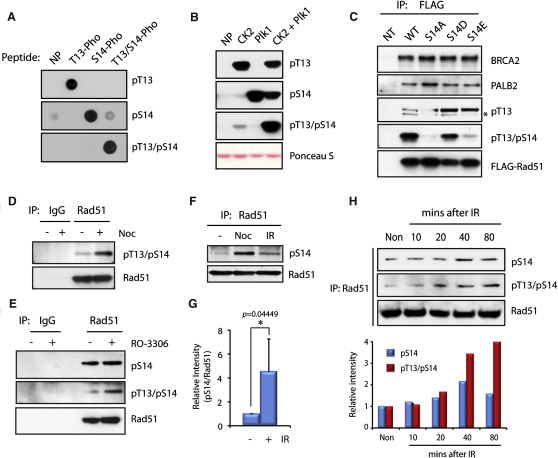
Rad51 Is Doubly Phosphorylated at T13 and S14 In Vivo (A) Synthetic Rad51 peptides with no phosphorylation (NP), phosphorylation at T13 (T13-Pho), S14 (S14-Pho), or both residues (T13/S14-Pho) were spotted on a nitrocellulose membrane and blotted with phospho-T13 antibody (pT13), phospho-S14 antibody (pS14), or diphospho-T13/S14 antibody (pT13/pS14). (B) Recombinant Rad51 was phosphorylated in vitro with CK2, Plk1, or both and detected with phospho-specific antibodies as above. Total protein was visualized by Ponceau S staining. (C) FLAG-tagged Rad51 variants purified from HEK293T were analyzed with either pT13, pT13/pS14, or FLAG antibody. Copurification of BRCA2 or PALB2 with FLAG-Rad51 is also shown. The asterisk indicates endogenous Rad51 copurified with FLAG-Rad51. (D) HeLa cells were treated with DMSO or nocodazole (Noc), and immunoprecipitated Rad51 was analyzed using the pT13/pS14 antibody. (E) HeLa cells were treated with DMSO or RO-3306, and Rad51 was analyzed with the pT13/pS14 or pS14 antibody. (F) HeLa cells were treated with nocodazole (Noc) or irradiated (IR, 4Gy). After 20 hr (Noc) or 20 min (IR) recovery, Rad51 was analyzed with the pS14 antibody. (G) Relative increase of S14 phosphorylated Rad51 at 20 min after irradiation is shown. Error bars, SD (n = 3); t test p value compared to nonirradiated cells is shown. Asterisk indicates t test p value < 0.05 (^∗^). (H) Top, HeLa cells were irradiated as above, and Rad51 phosphorylation was analyzed as above. Bottom, relative intensity of phosphorylated Rad51 against total Rad51 is shown.

The sequential phosphorylation of the Rad51 DTSV motif by Plk1 and CK2 in vitro prompted us to further investigate whether endogenous Rad51 is phosphorylated at T13 or at both T13 and S14 in cells. For this purpose, additional phospho-specific antibodies were raised against Rad51 monophosphorylated at T13 or diphosphorylated at T13 and S14 (pT13 and pT13/pS14 antibody, respectively; Figures 4A and 4B), and used to analyze in vivo phosphorylated Rad51. As shown in Figure 4C, FLAG-tagged WT Rad51 purified from HEK293T cells but not the S14A variant was detected both with the pT13 and the pT13/pS14 antibodies, showing that exogenously expressed Rad51 can be singly or doubly phosphorylated at these sites. pT13 antibody also detected increased signal in S14D/E variants, consistent with the modified CK2-mediated phosphorylation of the Rad51 S14 variants shown in Figure 3D. On the other hand, pT13/pS14 antibody detected only the Rad51 S14D substitution mutant, suggesting that this variant closely resembles doubly phosphorylated Rad51 when expressed in cells. BRCA2 and PALB2, known Rad51-binding partners, were efficiently copurified with all Rad51 variants in this system (Figure 4C), showing that S14 is not involved in the formation of the Rad51-BRCA2-PALB2 complex. To gain additional insight into the dynamics of Rad51 phosphorylation in vivo, we further investigated this process in HeLa cells. As was the case with S14 single phosphorylation, increased double phosphorylation of Rad51 was observed when cells were arrested in mitosis with nocodazole (Figure 4D). Additionally, when cells were arrested in G2 by blocking CDK1 activity with RO-3306 (Figures S2A and S2B) (Vassilev et al., 2006), increased T13/S14 doubly phosphorylated Rad51 was detected (Figure 4E). Because Rad51 plays a central role in DSB repair by HR, we next tested whether these sites are phosphorylated in response to IR. Strikingly, we found that S14 phosphorylation was transiently stimulated shortly after irradiation (20–40 min), followed by accumulation of double phosphorylation of Rad51 at T13/S14 (Figures 4F, 4G, 4H, S2C, S2D, and S2E).

### Phosphorylation-Dependent Rad51 Binding to Nbs1

**Figure 5:**
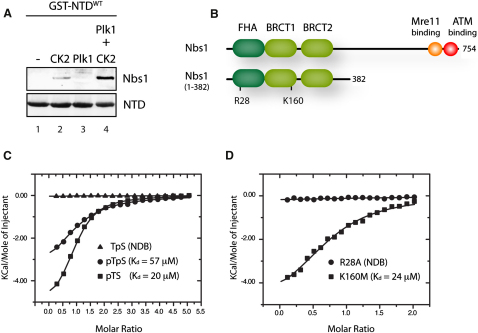
CK2-Phosphorylated Rad51 Interacts with Nbs1 (A) Rad51 NTD was phosphorylated with CK2 and/or Plk1, and Nbs1 interaction was analyzed by far-western blotting. (B) Schematic representation of Nbs1 and the Nbs1 (1-382) fragment used for the ITC experiments. (C) ITC titration of WT recombinant Nbs1 (1–382) with Rad51 NTD peptides phosphorylated at T13 (pTS), S14 (TpS), or T13/S14 (pTpS). NDB indicates nondetectable binding. (D) ITC titration of Nbs1 (1–382) containing mutation of the FHA domain (R28A) or the BRCT repeat domain (K160M) with the phospho-T13 Rad51 NTD peptide.

Given the close similarity between the Rad51 DTSV motif and Mdc1 SDTD motifs (Figure 3A), we next examined whether phosphorylation of Rad51 by Plk1 and/or CK2 triggers its interaction with Nbs1. Far-western blotting using recombinant full-length Nbs1 (a kind gift from Tanya Paull) revealed no interaction with nonphosphorylated or Plk1-phosphorylated Rad51, whereas increased interaction with CK2-phosphorylated Rad51 was observed (Figure 5A, lane 2). Notably, enhanced Nbs1-binding was detected when Rad51 was phosphorylated with both Plk1 and CK2 (Figure 5A, lane 4). A similar effect was observed when we used Rad51 NTD with S14D or S14E substitution but not S14A (Figures S3A and S3B).

To assess accurately the Rad51 phosphorylation status that mediates interaction with Nbs1, we further investigated the binding using isothermal titration calorimetry (ITC). Nbs1 contains two separate phospho-binding domains, namely the FHA domain and the BRCA1 C-terminus (BRCT) repeat domain, both of which interact with CK2-phosphorylated Mdc1 (Figure 5B) (Lloyd et al., 2009). Titrations of a recombinant fragment of Nbs1 (residues 1–382) encompassing the FHA and BRCT-repeat domains with either a T13 monophosphorylated Rad51 NTD peptide or a T13/S14 diphosphorylated version showed clear binding with affinities of around 20 μM and 50 μM, respectively, and stoichiometries that suggest binding to only one of the two potential Nbs1 phospho-binding domains (Figure 5C). In contrast, control titrations with either a nonphosphorylated Rad51 NTD peptide or one containing single S14 phosphorylation showed no detectable binding. We repeated the binding measurements using Nbs1 containing mutations that specifically disrupt FHA (R28A) or BRCT repeat (K160M) phospho-binding activity (Lloyd et al., 2009). Although binding of the phospho-T13 Rad51 NTD peptide to the K160M BRCT repeat domain mutant was maintained, no detectable binding to the R28A FHA domain mutant was observed (Figure 5D). Taken together, these data show that initial Plk1 phosphorylation of S14 serves mainly to prime CK2 phosphorylation at T13 and also that it is this second modification that is responsible for triggering Rad51 binding to the FHA domain of Nbs1.

### Roles of the Plk1 and CK2 Sites on Rad51 Following Genotoxic Stresses In Vivo

**Figure 6:**
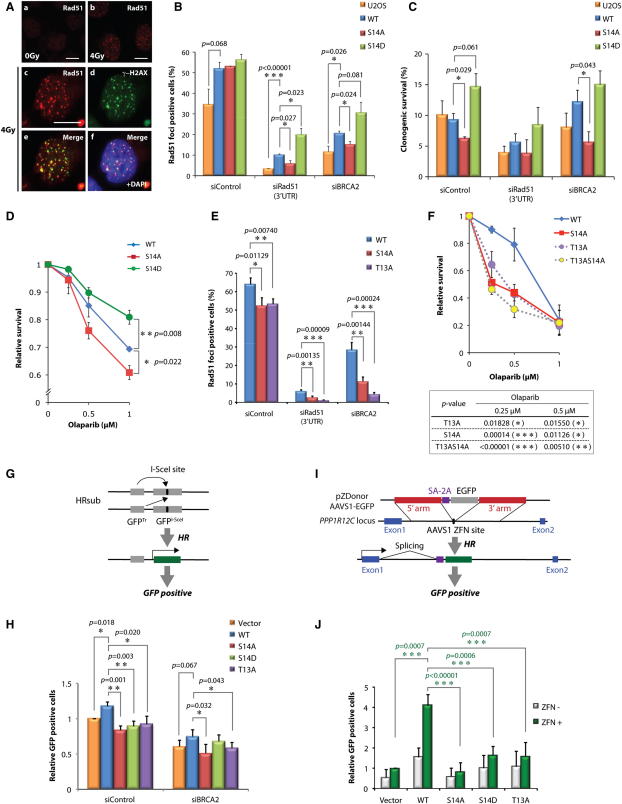
DNA Damage Response of Cells Expressing Rad51 Variants (A) U2OS cells expressing exogenous Rad51 were irradiated at 4 Gy. After 2.5 hr of recovery, Rad51 foci (a, b, and c) and γ−H2AX foci (d) were visualized by immunofluorescence staining. Colocalization of Rad51 and γ−H2AX foci is shown in (e) as a merged image of (c) and (d), and nuclear DNA staining with DAPI is shown in (f). The bars indicate 10 μm. (B) Cells expressing Rad51 variants at S14 were treated with indicated siRNA. Following 4 Gy irradiation, cells containing more than 20 Rad51 foci in a sample of > 150 cells were counted. Error bars, SD (n = 3). (C) Cell survival after IR (4 Gy) was examined by clonogenic assay. Error bars, SD (n = 3). (D) siBRCA2-treated cells expressing Rad51 variants at S14 were exposed to a PPAR inhibitor, Olaparib, for 4 days. Cell survival was assessed by WST-1 assay. Error bars, SD (n = 3). (E) Rad51 foci in cells expressing Rad51 variants at either S14 or T13 are analyzed as in (B). Error bars, SD (n = 3). (F) siBRCA2-treated cells expressing Rad51 variants at either S14 or T13 were exposed to the indicated dose of Olaparib for 5 days. Cell survival was assessed as above. Error bars, SD (n = 3). (G) Schematic representation depicting the inter-sister HR assay using tandem GFP substrates (HRsub). Active GFP is expressed when I-SceI-induced DSB in a mutant GFP^I-SceI^ is repaired by HR using truncated GFP^Tr^. Error bars, SD (n = 3). (H) Relative intensity of GFP signal following I-SceI expression is shown. Error bars, SD (n = 3). (I) Schematic representation depicting DSB-induced gene targeting. Promoterless GFP can be expressed from the *PPP1R12C* native promoter when the donor plasmid is targeted at the AAVS1 site by HR. (J) Relative intensity of GFP signal following pZDonor-AAVS1-GFP transfection is shown. Error bars, SD (n = 3). In panels (B), (C), (D), (E), (F), (H), and (J), t test p values compared to WT-expressing cells < 0.1 are shown. Asterisks indicate t test p value < 0.05 (^∗^), < 0.01 (^∗∗^) or < 0.001 (^∗∗∗^).

To examine further whether the S14 residue plays physiologically important roles, U2OS cell lines stably expressing nontagged versions of Rad51 S14 variants were generated (Figure S4A). Flow cytometry showed that the cell-cycle profiles of the stable cell lines were indistinguishable (Figure S4B). When cells were irradiated, the cell lines formed characteristic Rad51 foci (Figure 6A, a and b), and these IR-induced Rad51 foci colocalized with a DSB marker, γ−H2AX (Figure 6A, panels c–f). These observations indicate that the exogenously expressed Rad51 in these cell lines, although in excess, was functionally recruited to sites of damage. When endogenous Rad51 was downregulated using siRNA targeting the 3′UTR, a significantly reduced number of cells containing Rad51 foci was detected with cells expressing Rad51 S14A compared to WT, whereas Rad51 S14D-expressing cells exhibited increased numbers of Rad51 foci-positive cells peaking at 2.5 hr after irradiation (Figure 6B and S4C). These results show the importance of the Rad51 S14 residue in damage-induced focus formation.

Our biochemical analyses shown in Figure 5 provided evidence that sequential Rad51 phosphorylation within the DSTV motif stimulates its binding to a major DSB sensor protein, Nbs1, which accumulates on damaged chromatin in a BRCA2-independent manner. Hence we speculated that the differential recruitment of Rad51 S14 variants was likely to be a BRCA2-independent process. Indeed, the cells expressing exogenous WT Rad51 effectively formed Rad51 foci after BRCA2 downregulation, whereas those expressing Rad51 S14A exhibited reduced focus formation (Figures 6B and S4D). We further tested clonogenic survival of these cell lines after downregulating endogenous Rad51 or BRCA2. In otherwise unperturbed cells, all variants exhibited comparable survival (Figure S4E). By contrast, cells expressing WT or S14D-substituted Rad51 but not the S14A variant showed enhanced survival after IR treatment following BRCA2 downregulation (Figure 6C). These observations further confirm the importance of the S14 residue in resistance to IR, independently of BRCA2 function.

HR-defective cells, including *BRCA2*-defective cancer cells, exhibit profound sensitivity to inhibitors of poly(ADP-ribose) polymerase (PARP); hence, cancers harboring mutations in *BRCA2* can be treated effectively with PARP inhibitors (Bryant et al., 2005; Farmer et al., 2005). However, the genome of *BRCA2*-defective cancer cells is highly unstable and some cancers gain resistance to PARP inhibitors through de novo mutations (Edwards et al., 2008; Sakai et al., 2008). Given that Plk1 upregulation is often associated with malignancy (Strebhardt, 2010; Taylor and Peters, 2008), we addressed the potential role of Plk1-dependent Rad51 phosphorylation in PARP inhibitor resistance. Remarkably, where BRCA2 was downregulated, cells expressing WT and the phospho-mimetic S14D Rad51 variant exhibited significantly higher resistance to a PARP inhibitor compared to cells expressing S14A (Figure 6D). These results indicate that Plk1-mediated phosphorylation of Rad51 at S14 facilitates resistance to PARP inhibition.

We further addressed whether the cellular phenotypes associated with Rad51 S14 substitution are the direct result of altered phospho-T13-dependent binding to Nbs1. Indeed, cells expressing the T13A variant exhibited impaired IR-induced Rad51 focus formation (Figure 6E) and reduced resistance to the PARP inhibitor in BRCA2 downregulated cells (Figure 6F), similar to those expressing the S14A variant. Taken together, these observations support the notion that CK2 and Plk1 act in concert to regulate damage-induced Rad51 localization and resistance to genotoxic stresses.

### Roles of the Plk1 and CK2 Sites on Rad51 in Homologous Recombination

Finally, we investigated the importance of the CK2 and Plk1 sites on Rad51 during HR in vivo. To this end, we used a well-established HR reporter system based on the rare-cutting homing endonuclease I-SceI to introduce a DSB (Moynahan and Jasin, 2010). Specifically, Rad51 variants were stably expressed in a U2OS cell line that carries tandem modified GFP genes on chromosome 18: a GFP mutant containing an I-SceI cleavage site (GFP^I-SceI^) and a truncated GFP (GFP^Tr^) (U2OS-SCR18) (Puget et al., 2005). Following I-SceI expression, HR events were measured by quantifying the GFP-expressing cell population (Figures 6G and S4F). As shown in Figure 6H, increased HR events were detected in cells expressing WT Rad51 compared to those with empty vector, whereas no increase was found with Rad51 variants at S14 and T13. Similar phenotypes were also observed following BRCA2 downregulation, although S14D supported modest HR recovery.

We further assessed HR in the absence of functional BRCA2 by DSB-mediated gene targeting in *BRCA2*-defective EUFA423 cells stably expressing Rad51 variants. We exploited a zinc-finger nuclease (ZFN) that introduces a DSB at a native AAVS1 locus within the *PPP1R12C* gene and a donor plasmid containing a promoterless GFP gene between sequences homologous to those flanking the AAVS1 site (Figures 6I, S4G, and S4H) (Brunet et al., 2009; Hockemeyer et al., 2009). In this system, DSB-promoted gene targeting results in GFP expression from the native *PPP1R12C* promoter. Indeed, a clear increase of GFP expression was observed in EUFA423 cells expressing WT Rad51 compared to those containing empty vector (Figure 6J). By contrast, EUFA423 cells expressing Rad51 variants at either T13 or S14 exhibited significantly lower targeting efficiency. These observations support the notion that dynamic phosphorylation of Rad51 by Plk1 and CK2 is important for the coordination of precise recombination.

## Discussion

**Figure 7:**
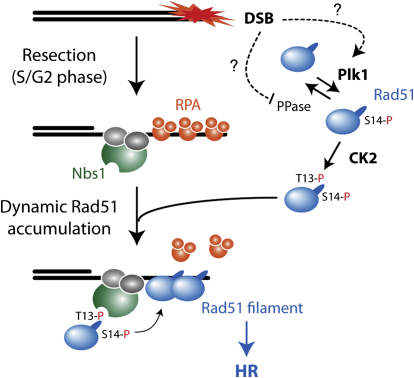
Model for Rad51 Recruitment Mediated through Sequential Phosphorylation by Plk1 and CK2 A model for Plk1-mediated Rad51 recruitment. See text for description.

In this study, we show that 1) Rad51 recombinase is directly phosphorylated by Plk1 at S14 in a cell cycle- and DNA damage-responsive manner; 2) Plk1-mediated phosphorylation stimulates subsequent CK2-mediated phosphorylation at T13; 3) T13 phosphorylation of Rad51 by CK2 triggers a direct interaction with the FHA domain of the MRN component, Nbs1; and 4) Rad51 phosphorylation at either S14 or T13 is important for accurate HR and for cellular resistance to IR and to PPAR inhibition. Collectively, these data support the model illustrated in Figure 7. Upon DSB induction, the MRN complex is efficiently recruited to the site of damage, mediating DNA resection during S and G2 phases of the cell cycle. During these cell-cycle phases, Plk1-mediated Rad51 phosphorylation increases, stimulating CK2-dependent T13 phosphorylation and triggering its interaction with the FHA domain of Nbs1. This mechanism helps increase the Rad51 concentration at the site of DNA damage and facilitates HR.

### Early Roles of Plk1 after DNA Damage

Mounting evidence points toward active roles of Plk1 in DNA damage responses (Macůrek et al., 2008; Syljuåsen et al., 2006; Toczyski et al., 1997; van Vugt et al., 2004; Yoo et al., 2004) but there is no established role for Plk1 immediately after DNA damage. Unexpectedly, we observed an immediate and transient increase of Rad51 phosphorylation after DNA damage: S14 phosphorylation reproducibly peaked at 20–40 min after irradiation (Figures 4F, 4G, 4H, S2C, S2D, and S2E). It is not clear whether total Plk1 activity is stimulated immediately after DNA damage or whether Plk1 becomes locally activated at the site of DNA damage. The latter possibility is particularly attractive in light of the observed interaction of the Plk1 PBD with damage-responsive proteins (e.g., Mdc1 [Lowery et al., 2007]) and the stimulatory effect on Plk1 activity on phospho-dependent interactions mediated through the PBD (Elia et al., 2003). Alternatively, it is also conceivable that S14-phosphorylated Rad51 increases because active Plk1 accumulates during cell-cycle progression, whereas DNA damage may block activity of a still unidentified Rad51 S14 phosphatase (see Figure 7).

### Molecular Roles of Plk1- and CK2-Mediated Rad51 Phosphorylation

Despite the emerging importance of CK2-mediated phosphorylation in DNA damage signaling (Ayoub et al., 2008; Chapman and Jackson, 2008; Loizou et al., 2004; Melander et al., 2008; Spycher et al., 2008), the molecular mechanism by which CK2 is regulated in response to DNA damage is largely unknown. Our study establishes a mechanism of indirect regulation whereby DNA damage-responsive Plk1 phosphorylation of Rad51 enables CK2 to modify a second site that is the main switch for binding to the effector, Nbs1. Although our ITC analysis indicates that T13 single phosphorylation showed strongest binding to Nbs1 among the peptides tested, a signal from endogenous Rad51 was not detectable with our pT13 antibody. This observation might be due to the quality and the titer of pT13 antibody but, given that T13 phosphorylation is highly stimulated upon S14 phosphorylation, we favor the idea that T13 phosphorylation happens mainly or only when the S14 site is phosphorylated in a physiological context.

This phospho-dependent interaction between Nbs1 and Rad51 also reveals a direct link between DNA damage recognition and HR repair. It is noteworthy that Nbs1 is recruited not only to chromatin, through interactions between the Nbs1 FHA domain and CK2-phosphorylated Mdc1 but also directly to ssDNA regions (Bekker-Jensen et al., 2006; Chapman and Jackson, 2008; Stracker and Petrini, 2011). The FHA domain of Nbs1, which is localized to ssDNA as part of the MRN complex, presumably is free from Mdc1 binding and may help recruit Rad51 to ssDNA. The modest affinities observed by ITC suggest that the interaction is likely to be rather dynamic, consistent with the fact that it was not detected by pull-down methodologies under the conditions we employed. We propose that the transient and dynamic interaction may help to increase Rad51 concentration at the site of DNA damage without anchoring Rad51 to Nbs1, which might otherwise block the formation of active Rad51 nucleoprotein filaments. Given that Rad51 assembles cooperatively onto DNA (Baumann et al., 1996), its local concentration may impact significantly on the initiation of this polymerization and, hence, HR itself. In parallel, BRCA2 that interacts with T13-phosphorylated Rad51 may also be jointly recruited through this mechanism and facilitate stable loading of Rad51 onto ssDNA.

### Roles of Plk1-Mediated Rad51 Phosphorylation in Unperturbed Cells

We found no evidence for a role of cell cycle-dependent Rad51 phosphorylation by Plk1 in unperturbed U2OS cells (Figures S4B and S4E), although it is possible that such phosphorylation may have a role in primary cells. Supporting this idea, active and protective roles of Rad51 in later phases of the cell cycle were shown using chicken DT40 and *Xenopus* systems; Rad51 depletion caused G2 arrest with accumulated ssDNA lesions or DSBs (Hashimoto et al., 2010; Su et al., 2008). In this context, Plk1-mediated Rad51 phosphorylation in G2 may promote its recruitment to a ssDNA lesion, providing a final opportunity to complete sister chromatid synthesis before onset of mitosis. This mechanism may also play a crucial role during meiosis, where HR facilitates crossover between homologous chromosomes. In line with this notion, a direct role of Cdc5, which is a yeast ortholog of Plk1, during meiotic crossover was recently reported (Matos et al., 2011). Further studies will be needed to fully illuminate Plk1-dependent HR regulation in somatic and germline cells.

### A Model for Genome Instability Phenotypes Mediated through Rad51 Phosphorylation

In HR reporter assays using site-specific endonucleases, Rad51 variants at the CK2 or Plk1 sites that were substituted with either nonphosphorylatable alanine (S14A and T13A) or phospho-mimetic aspartate (S14D) were less proficient in HR than WT Rad51. This observation suggests that both impaired and unduly stable interaction between Rad51 and Nbs1 have negative effects on canonical error-free HR, highlighting the importance of dynamic quality control of HR proteins during DSB repair (Kanaar et al., 2008). Intriguingly, the S14D variant exhibited increased survival following IR and Olaparib treatment (Figures 6C and 6D), leading us to propose that enhanced Rad51-binding to Nbs1 may support survival by promoting nonlethal but low quality recombination events that were undetectable with the HR reporter systems used in this study. This idea is supported by the observation that elevated Rad51 expression in mouse embryonic stem cells leads to aberrant interchromosomal repair following induction of multiple DSBs within short homologous sequences, consequently resulting in a genome instability phenotype (Richardson et al., 2004). We also found that BRCA2-defective EUFA423 cells exhibited increased random integration of donor plasmid when WT Rad51 was exogenously expressed (Figures 6J, S4G, and S4H). Therefore, Plk1-mediated Rad51 phosphorylation may promote gross chromosomal instability, particularly when fine-tuning of HR is disrupted in *BRCA*-defective cells. Importantly, increased activity of Plk1 is closely linked to malignancy, and Plk1 inhibition sensitizes cancer cells to DNA damage treatment (Strebhardt, 2010; Sur et al., 2009; Taylor and Peters, 2008). Clinical trials in cancer patients are currently underway to evaluate the effects of PARP and Plk1 inhibitors (Carden et al., 2010; Lord and Ashworth, 2008). It is tempting to speculate that a combined therapy using inhibitors of both Plk1 and PARP may be an effective approach to improve prognosis of *BRCA*-defective cancers.

In summary, we have established a direct link between DNA damage recognition and HR repair, mediated through a phospho-dependent interaction between Nbs1 and Rad51. A primary cell-cycle regulator, Plk1, plays a critical role in the regulation of this interaction, which can modulate the BRCA2 dependency of HR repair. Our findings represent a significant step toward a comprehensive understanding of HR regulation by cell-cycle regulators, which may be exploited in the further development of effective cancer treatments.

## Experimental Procedures

### Cell Culture

HeLa, HEK293T, and U2OS cells were cultured at 37°C with 5% CO_2_ in Dulbecco's modified Eagle's medium supplemented with streptomycin (0.1 mg/ml), penicillin (100 units/ml), and 10% v/v fetal bovine serum. Where indicated, cells were treated with 0.2 mM nocodazole (Sigma-Aldrich) or 9 μM RO-3306 (Enzo Life Sciences) for 20 hr, or 50 μM BTO-1 (Sigma-Aldrich) or 0.1 μM BI-2536 (Axon Medchem) for 2.5 hr. The ^137^Cs-source of an IBL 637 (CIS Bio International; Figures 4F, 4G, S2C, and S2D) or a GRAVITRON RX 30/55 (Gravatom; Figures 4H and 6) was used to irradiate cells at 4 Gy (59 s and 69 s, respectively). Cell synchronization was carried out as previously described (Esashi et al., 2005). U2OS stable cell lines expressing Rad51 variants were generated by cotransfecting pcDNA5/FRT encoding nontagged Rad51 and pcDNA-DEST26 or pcDNA-DEST53 (Invitrogen) using jetPrime (Polyplus Transfection), followed by G418 selection at 400 μg/ml. U2OS-SCR18 was a kind gift from Ralph Scully. U2OS-SCR18 cells expressing Rad51 variants were generated by transfecting pT-Rex-DEST30 encoding nontagged Rad51, followed by selection with 300 μg/ml G418 and 1 μg/ml puromycin. EUFA423 cell lines stably expressing Rad51 variants were generated in two steps: EUFA423 cells were transfected with pFRT/lacZeo plasmid (Invitrogen), and a cell line containing a Flp-In recombination site was cloned following Zeocin selection at 25 μg/ml. Established EUFA423 Flp-In cells were then used to generate stable cell lines by cotransfecting pOG44 (Invitrogen) and nontagged Rad51 variant in pcDNA5/FRT, followed by hygromycin selection at 50 μg/ml. For siRNA treatments, cells were seeded at a density of 1.5 × 10^5^ cells in 6-well plates and then transfected, on the following day, with siRad51 (20 nM), siBRCA2 (100 nM), or control siRNA with DharmaFECT1 (100 nM) (Dharmacon); cells were further incubated for 24 hr before analyses.

### Extract Preparation, Immunoprecipitation, and Western Blotting

Cell extract was prepared using extraction buffer (150 mM KCl, 20 mM HEPES pH7.6, 2 mM EGTA, 1.5 mM MgCl_2_, 50 mM NaF, 0.1% NP40, 10% glycerol, 1 mM Na_3_VO_4_, 20 mM β-glycerophosphate, 1 mM dithiothreitol, 10 mM benzamidine HCl, 25 units/ml Benzonase nuclease [Novagen]) supplemented with Protease inhibitor cocktail (Sigma-Aldrich, P2714). For immunoprecipitation, the extract was precleared with 10 μl of control IgG beads, followed by incubation with antibody cross-linked to beads. After extensive washing, immune complexes were separated by SDS-PAGE and analyzed by western blotting, following standard protocols. Where indicated, the membrane was treated with Re-Blot Plus Mild Solution (Millipore) before incubating with another antibody; for Rad51 phosphorylation analyses, pT13/pS14, pS14, and Rad51 antibodies were applied in this order.

### In Vitro Kinase Reactions and Far-Western Blotting

Protein substrates (1 μg in 15 μl total volume) were phosphorylated in kinase buffer (25 mM MOPS pH 7.2, 25 mM β-glycerophosphate, 15 mM MgCl_2_, 1% DMSO, 7.5 μM ATP, 1 mM DTT and 1 μCi γ^32^P-ATP) by the addition of 1 μl of recombinant Plk1 or CK2. Following incubation at 30°C for 30 min, reactions were stopped by heating at 95°C for 5 min in SDS sample buffer. Proteins were then resolved by SDS-PAGE and visualized by staining with InstantBlue (Expedeon). After drying gel with DryEase (Invitrogen), ^32^P-labeled products were detected by autoradiography. For far-western analysis, kinase reaction was carried out in kinase buffer supplemented with 250 μM ATP, with no γ^32^P-ATP. The reaction mixture was then resolved by SDS-PAGE and transferred to a Protran nitrocellulose membrane (Whatman, BA85), followed by incubation with recombinant full-length FLAG-Nbs1 (∼ 2 μg). Anti-Nbs1 antibody was then applied to the membrane to detect Nbs1 protein.

### Isothermal Titration Calorimetry

The affinities and thermodynamic parameters for human Nbs1-Rad51 phosphopeptide interactions were determined by isothermal titration calorimetry with a ITC-200 instrument (MicroCal) at 18°C. Protein samples were dialyzed extensively into 50 mM HEPES (pH 7.5), 150 mM NaCl, 2 mM β-mercaptoethanol and peptides were desalted and buffer exchanged using NAP-5 purification columns (GE Healthcare) into the relevant buffer (Lloyd et al., 2009). In general, peptides (0.5–1.5 mM) were titrated into 0.05–0.1 mM Nbs1. Peptides were synthesized by W. Mawby (University of Bristol) and their composition was verified by mass spectrometry. Data were analyzed with Origin 7.0 software.

### Cell Survival Assay

For clonogenic assay, U2OS cells stably expressing Rad51 variants were treated with siRNA for 24 hr before plating at a density of 500 or 5,000 cells in a 100 mm plate. At 48 hr after siRNA transfection, plates seeded with 5,000 cells were irradiated at 4 Gy from a ^137^Cs-source of a GRAVITRON RX 30/55 irradiator (Gravatom) and incubated further for 14 days. Cells were then fixed and stained with Coomassie stain, and colonies of > 50 cells were counted. The mean surviving fraction was calculated as a percentage of the mean seen in the nonirradiated control. To assess sensitivity to the PARP1 inhibitor Olaparib, cells treated with siRNA were seeded at a density of 5,000 cells (for Figure 6D) or 1,500 cells (for Figure 6F) in 96-well plates at 24 hr after siRNA transfection. Once cells had adhered to the plate, Olaparib/AZD 2281 (Axon Medchem) was added at the indicated concentration. For Figure 6F, medium containing Olaparib or vehicle was replenished at 48 hr and cells were further incubated for 72 hr (Turner et al., 2008). Cell survival relative to vehicle-treated cells was then assessed using the WST-1 kit (Roche) according to the manufacturer's protocol.

### In Vivo Recombination Assay Using Site-Specific Endonucleases

HR assay using U2OS-SCR18 was performed as described previously (Puget at al., 2005). For HR-mediated gene targeting assay, EUFA423 Flp-In cells stably expressing Rad51 variants were seeded at a density of 1.9 × 10^5^ per 6-wells, and transfected 24 hr later with pZDonor-AAVS1-SA-2P-GFP (800 μg) (DeKelver et al., 2010; Hockemeyer et al., 2009) with or without 100 ng of each AAVS1 zinc-finger nuclease (ZFN) encoding plasmids (pZFN1-AAVS1L and pZFN2-AAVS1R). Four hours posttransfection, cells were trypsinized and reseeded on a 10 cm dish and further incubated for 4 days. Frequency of GFP-positive cells was quantified by FACS using a FACSCalibur flow cytometer and analyzed on a green (FL1 channel) against red (FL2 channel) autofluorescence plot with CellQuest Pro software (Becton Dickinson).

## Supplemental Information

Document S1. Supplemental Experimental Procedures and Figures S1–S4

## References

[bib1] Alexander J., Lim D., Joughin B.A., Hegemann B., Hutchins J.R.A., Ehrenberger T., Ivins F., Sessa F., Hudecz O., Nigg E.A. (2011). Spatial exclusivity combined with positive and negative selection of phosphorylation motifs is the basis for context-dependent mitotic signaling. Sci. Signal..

[bib2] Ayoub N., Jeyasekharan A.D., Bernal J.A., Venkitaraman A.R. (2008). HP1-beta mobilization promotes chromatin changes that initiate the DNA damage response. Nature.

[bib3] Barr F.A., Silljé H.H., Nigg E.A. (2004). Polo-like kinases and the orchestration of cell division. Nat. Rev. Mol. Cell Biol..

[bib4] Baumann P., Benson F.E., West S.C. (1996). Human Rad51 protein promotes ATP-dependent homologous pairing and strand transfer reactions in vitro. Cell.

[bib5] Bekker-Jensen S., Lukas C., Kitagawa R., Melander F., Kastan M.B., Bartek J., Lukas J. (2006). Spatial organization of the mammalian genome surveillance machinery in response to DNA strand breaks. J. Cell Biol..

[bib6] Brown E.T., Holt J.T. (2009). Rad51 overexpression rescues radiation resistance in BRCA2-defective cancer cells. Mol. Carcinog..

[bib7] Brunet E., Simsek D., Tomishima M., DeKelver R., Choi V.M., Gregory P., Urnov F., Weinstock D.M., Jasin M. (2009). Chromosomal translocations induced at specified loci in human stem cells. Proc. Natl. Acad. Sci. USA.

[bib8] Bryant H.E., Schultz N., Thomas H.D., Parker K.M., Flower D., Lopez E., Kyle S., Meuth M., Curtin N.J., Helleday T. (2005). Specific killing of BRCA2-deficient tumours with inhibitors of poly(ADP-ribose) polymerase. Nature.

[bib9] Carden C.P., Yap T.A., Kaye S.B. (2010). PARP inhibition: targeting the Achilles' heel of DNA repair to treat germline and sporadic ovarian cancers. Curr. Opin. Oncol..

[bib10] Chapman J.R., Jackson S.P. (2008). Phospho-dependent interactions between NBS1 and MDC1 mediate chromatin retention of the MRN complex at sites of DNA damage. EMBO Rep..

[bib11] Connor F., Bertwistle D., Mee P.J., Ross G.M., Swift S., Grigorieva E., Tybulewicz V.L., Ashworth A. (1997). Tumorigenesis and a DNA repair defect in mice with a truncating Brca2 mutation. Nat. Genet..

[bib12] DeKelver R.C., Choi V.M., Moehle E.A., Paschon D.E., Hockemeyer D., Meijsing S.H., Sancak Y., Cui X.X., Steine E.J., Miller J.C. (2010). Functional genomics, proteomics, and regulatory DNA analysis in isogenic settings using zinc finger nuclease-driven transgenesis into a safe harbor locus in the human genome. Genome Res..

[bib13] Digweed M., Reis A., Sperling K. (1999). Nijmegen breakage syndrome: consequences of defective DNA double strand break repair. Bioessays.

[bib14] Edwards S.L., Brough R., Lord C.J., Natrajan R., Vatcheva R., Levine D.A., Boyd J., Reis-Filho J.S., Ashworth A. (2008). Resistance to therapy caused by intragenic deletion in BRCA2. Nature.

[bib15] Elia A.E., Rellos P., Haire L.F., Chao J.W., Ivins F.J., Hoepker K., Mohammad D., Cantley L.C., Smerdon S.J., Yaffe M.B. (2003). The molecular basis for phosphodependent substrate targeting and regulation of Plks by the Polo-box domain. Cell.

[bib16] Esashi F., Christ N., Gannon J., Liu Y., Hunt T., Jasin M., West S.C. (2005). CDK-dependent phosphorylation of BRCA2 as a regulatory mechanism for recombinational repair. Nature.

[bib17] Farmer H., McCabe N., Lord C.J., Tutt A.N., Johnson D.A., Richardson T.B., Santarosa M., Dillon K.J., Hickson I., Knights C. (2005). Targeting the DNA repair defect in BRCA mutant cells as a therapeutic strategy. Nature.

[bib18] Hashimoto Y., Chaudhuri A.R., Lopes M., Costanzo V. (2010). Rad51 protects nascent DNA from Mre11-dependent degradation and promotes continuous DNA synthesis. Nat. Struct. Mol. Biol..

[bib19] Hockemeyer D., Soldner F., Beard C., Gao Q., Mitalipova M., DeKelver R.C., Katibah G.E., Amora R., Boydston E.A., Zeitler B. (2009). Efficient targeting of expressed and silent genes in human ESCs and iPSCs using zinc-finger nucleases. Nat. Biotechnol..

[bib20] Huertas P., Cortés-Ledesma F., Sartori A.A., Aguilera A., Jackson S.P. (2008). CDK targets Sae2 to control DNA-end resection and homologous recombination. Nature.

[bib21] Ira G., Pellicioli A., Balijja A., Wang X., Fiorani S., Carotenuto W., Liberi G., Bressan D., Wan L., Hollingsworth N.M. (2004). DNA end resection, homologous recombination and DNA damage checkpoint activation require CDK1. Nature.

[bib22] Jazayeri A., Falck J., Lukas C., Bartek J., Smith G.C., Lukas J., Jackson S.P. (2006). ATM- and cell cycle-dependent regulation of ATR in response to DNA double-strand breaks. Nat. Cell Biol..

[bib23] Jensen R.B., Carreira A., Kowalczykowski S.C. (2010). Purified human BRCA2 stimulates RAD51-mediated recombination. Nature.

[bib24] Kanaar R., Wyman C., Rothstein R. (2008). Quality control of DNA break metabolism: in the ‘end’, it's a good thing. EMBO J..

[bib25] Lancaster J.M., Wooster R., Mangion J., Phelan C.M., Cochran C., Gumbs C., Seal S., Barfoot R., Collins N., Bignell G. (1996). BRCA2 mutations in primary breast and ovarian cancers. Nat. Genet..

[bib26] Lee S.A., Roques C., Magwood A.C., Masson J.Y., Baker M.D. (2009). Recovery of deficient homologous recombination in Brca2-depleted mouse cells by wild-type Rad51 expression. DNA Repair (Amst.).

[bib27] Liu J., Doty T., Gibson B., Heyer W.D. (2010). Human BRCA2 protein promotes RAD51 filament formation on RPA-covered single-stranded DNA. Nat. Struct. Mol. Biol..

[bib28] Lloyd J., Chapman J.R., Clapperton J.A., Haire L.F., Hartsuiker E., Li J.J., Carr A.M., Jackson S.P., Smerdon S.J. (2009). A supramodular FHA/BRCT-repeat architecture mediates Nbs1 adaptor function in response to DNA damage. Cell.

[bib29] Loizou J.I., El-Khamisy S.F., Zlatanou A., Moore D.J., Chan D.W., Qin J., Sarno S., Meggio F., Pinna L.A., Caldecott K.W. (2004). The protein kinase CK2 facilitates repair of chromosomal DNA single-strand breaks. Cell.

[bib30] Lord C.J., Ashworth A. (2008). Targeted therapy for cancer using PARP inhibitors. Curr. Opin. Pharmacol..

[bib31] Lowery D.M., Lim D., Yaffe M.B. (2005). Structure and function of Polo-like kinases. Oncogene.

[bib32] Lowery D.M., Clauser K.R., Hjerrild M., Lim D., Alexander J., Kishi K., Ong S.E., Gammeltoft S., Carr S.A., Yaffe M.B. (2007). Proteomic screen defines the Polo-box domain interactome and identifies Rock2 as a Plk1 substrate. EMBO J..

[bib33] Macůrek L., Lindqvist A., Lim D., Lampson M.A., Klompmaker R., Freire R., Clouin C., Taylor S.S., Yaffe M.B., Medema R.H. (2008). Polo-like kinase-1 is activated by aurora A to promote checkpoint recovery. Nature.

[bib34] Matos J., Blanco M.G., Maslen S., Skehel J.M., West S.C. (2011). Regulatory control of the resolution of DNA recombination intermediates during meiosis and mitosis. Cell.

[bib35] Melander F., Bekker-Jensen S., Falck J., Bartek J., Mailand N., Lukas J. (2008). Phosphorylation of SDT repeats in the MDC1 N terminus triggers retention of NBS1 at the DNA damage-modified chromatin. J. Cell Biol..

[bib36] Moynahan M.E., Jasin M. (2010). Mitotic homologous recombination maintains genomic stability and suppresses tumorigenesis. Nat. Rev. Mol. Cell Biol..

[bib37] Puget N., Knowlton M., Scully R. (2005). Molecular analysis of sister chromatid recombination in mammalian cells. DNA Repair (Amst.).

[bib38] Qing Y., Yamazoe M., Hirota K., Dejsuphong D., Sakai W., Yamamoto K.N., Bishop D.K., Wu X., Takeda S. (2011). The epistatic relationship between BRCA2 and the other RAD51 mediators in homologous recombination. PLoS Genet..

[bib39] Rass E., Grabarz A., Plo I., Gautier J., Bertrand P., Lopez B.S. (2009). Role of Mre11 in chromosomal nonhomologous end joining in mammalian cells. Nat. Struct. Mol. Biol..

[bib40] Richardson C., Stark J.M., Ommundsen M., Jasin M. (2004). Rad51 overexpression promotes double-strand break repair pathways and genome instability. Oncogene.

[bib41] Sakai W., Swisher E.M., Karlan B.Y., Agarwal M.K., Higgins J., Friedman C., Villegas E., Jacquemont C., Farrugia D.J., Couch F.J. (2008). Secondary mutations as a mechanism of cisplatin resistance in BRCA2-mutated cancers. Nature.

[bib42] Sarno S., Vaglio P., Marin O., Issinger O.G., Ruffato K., Pinna L.A. (1997). Mutational analysis of residues implicated in the interaction between protein kinase CK2 and peptide substrates. Biochemistry.

[bib43] Songyang Z., Lu K.P., Kwon Y.T., Tsai L.H., Filhol O., Cochet C., Brickey D.A., Soderling T.R., Bartleson C., Graves D.J. (1996). A structural basis for substrate specificities of protein Ser/Thr kinases: primary sequence preference of casein kinases I and II, NIMA, phosphorylase kinase, calmodulin-dependent kinase II, CDK5, and Erk1. Mol. Cell. Biol..

[bib44] Spycher C., Miller E.S., Townsend K., Pavic L., Morrice N.A., Janscak P., Stewart G.S., Stucki M. (2008). Constitutive phosphorylation of MDC1 physically links the MRE11-RAD50-NBS1 complex to damaged chromatin. J. Cell Biol..

[bib45] Stankiewicz P., Lupski J.R. (2002). Genome architecture, rearrangements and genomic disorders. Trends Genet..

[bib46] Stracker T.H., Petrini J.H. (2011). The MRE11 complex: starting from the ends. Nat. Rev. Mol. Cell Biol..

[bib47] Strebhardt K. (2010). Multifaceted polo-like kinases: drug targets and antitargets for cancer therapy. Nat. Rev. Drug Discov..

[bib48] Su X., Bernal J.A., Venkitaraman A.R. (2008). Cell-cycle coordination between DNA replication and recombination revealed by a vertebrate N-end rule degron-Rad51. Nat. Struct. Mol. Biol..

[bib49] Sur S., Pagliarini R., Bunz F., Rago C., Diaz L.A., Kinzler K.W., Vogelstein B., Papadopoulos N. (2009). A panel of isogenic human cancer cells suggests a therapeutic approach for cancers with inactivated p53. Proc. Natl. Acad. Sci. USA.

[bib50] Syljuåsen R.G., Jensen S., Bartek J., Lukas J. (2006). Adaptation to the ionizing radiation-induced G2 checkpoint occurs in human cells and depends on checkpoint kinase 1 and Polo-like kinase 1 kinases. Cancer Res..

[bib51] Takaki T., Trenz K., Costanzo V., Petronczki M. (2008). Polo-like kinase 1 reaches beyond mitosis—cytokinesis, DNA damage response, and development. Curr. Opin. Cell Biol..

[bib52] Tarsounas M., Davies D., West S.C. (2003). BRCA2-dependent and independent formation of RAD51 nuclear foci. Oncogene.

[bib53] Tauchi H., Kobayashi J., Morishima K., van Gent D.C., Shiraishi T., Verkaik N.S., vanHeems D., Ito E., Nakamura A., Sonoda E. (2002). Nbs1 is essential for DNA repair by homologous recombination in higher vertebrate cells. Nature.

[bib54] Taylor S., Peters J.M. (2008). Polo and Aurora kinases: lessons derived from chemical biology. Curr. Opin. Cell Biol..

[bib55] Thorslund T., McIlwraith M.J., Compton S.A., Lekomtsev S., Petronczki M., Griffith J.D., West S.C. (2010). The breast cancer tumor suppressor BRCA2 promotes the specific targeting of RAD51 to single-stranded DNA. Nat. Struct. Mol. Biol..

[bib56] Toczyski D.P., Galgoczy D.J., Hartwell L.H. (1997). CDC5 and CKII control adaptation to the yeast DNA damage checkpoint. Cell.

[bib57] Turner N.C., Lord C.J., Iorns E., Brough R., Swift S., Elliott R., Rayter S., Tutt A.N., Ashworth A. (2008). A synthetic lethal siRNA screen identifying genes mediating sensitivity to a PARP inhibitor. EMBO J..

[bib58] Tutt A., Connor F., Bertwistle D., Kerr P., Peacock J., Ross G., Ashworth A. (2003). Cell cycle and genetic background dependence of the effect of loss of BRCA2 on ionizing radiation sensitivity. Oncogene.

[bib59] van Gent D.C., Hoeijmakers J.H., Kanaar R. (2001). Chromosomal stability and the DNA double-stranded break connection. Nat. Rev. Genet..

[bib60] van Vugt M.A., Brás A., Medema R.H. (2004). Polo-like kinase-1 controls recovery from a G2 DNA damage-induced arrest in mammalian cells. Mol. Cell.

[bib61] Varon R., Vissinga C., Platzer M., Cerosaletti K.M., Chrzanowska K.H., Saar K., Beckmann G., Seemanová E., Cooper P.R., Nowak N.J. (1998). Nibrin, a novel DNA double-strand break repair protein, is mutated in Nijmegen breakage syndrome. Cell.

[bib62] Vassilev L.T., Tovar C., Chen S., Knezevic D., Zhao X., Sun H., Heimbrook D.C., Chen L. (2006). Selective small-molecule inhibitor reveals critical mitotic functions of human CDK1. Proc. Natl. Acad. Sci. USA.

[bib63] Venkitaraman A.R. (2002). Cancer susceptibility and the functions of BRCA1 and BRCA2. Cell.

[bib64] West S.C. (2003). Molecular views of recombination proteins and their control. Nat. Rev. Mol. Cell Biol..

[bib65] Williams R.S., Dodson G.E., Limbo O., Yamada Y., Williams J.S., Guenther G., Classen S., Glover J.N., Iwasaki H., Russell P., Tainer J.A. (2009). Nbs1 flexibly tethers Ctp1 and Mre11-Rad50 to coordinate DNA double-strand break processing and repair. Cell.

[bib66] Wyman C., Kanaar R. (2004). Homologous recombination: down to the wire. Curr. Biol..

[bib67] Xie A., Kwok A., Scully R. (2009). Role of mammalian Mre11 in classical and alternative nonhomologous end joining. Nat. Struct. Mol. Biol..

[bib68] Yoo H.Y., Kumagai A., Shevchenko A., Shevchenko A., Dunphy W.G. (2004). Adaptation of a DNA replication checkpoint response depends upon inactivation of Claspin by the Polo-like kinase. Cell.

[bib69] Yun M.H., Hiom K. (2009). CtIP-BRCA1 modulates the choice of DNA double-strand-break repair pathway throughout the cell cycle. Nature.

