# Supplementary material for: Plk1 and CK2 Act in Concert to Regulate Rad51 during DNA Double Strand Break Repair
Source: Mol Cell. 2012 Feb 10;45(3):371–83. doi: 10.1016/j.molcel.2011.12.028 (PMC3280358; doi:10.1016/j.molcel.2011.12.028)
Supplement: Document S1. Supplemental Experimental Procedures and Figures S1–S4 [file mmc1.pdf]

Molecular Cell, ***Volume 45***

Supplemental Information

Plk1 and CK2 Act in Concert to Regulate Rad51

during DNA Double Strand Break Repair

Keiko Yata, Janette Lloyd, Sarah Maslen, Jean-Yves Bleuyard, Mark Skehel, Stephen J. Smerdon, and Fumiko Esashi

SUPPLEMENTAL EXPERIMENTAL PROCEDURES

Plasmids and Proteins

Rad51 point mutations were introduced using QuikChange Site-Directed Mutagenesis Kit (Stratagene) according to the manufacturer's protocol, and were confirmed by DNA sequencing. For mammalian expression, human Rad51 was cloned into pcDNA5/FRT vector (Invitrogen) with or without FLAG epitope at N-terminus. Rad51 NTD (1-86) variants were PCR cloned into pET11d (Stratagene) or pGEX-4T-3 (GE Healthcare), and the recombinant proteins were purified following standard protocols using TALON Metal Affinity Resin (Clontech) or Glutathione Sepharose (GE Healthcare). Recombinant full-length Rad51 (Baumann et al., 1997), ATPase core Rad51 (Esashi et al., 2007), and Nbs1 (1-382) fragments (Lloyd et al., 2009) were prepared as described previously. Recombinant full-length FLAG-Nbs1 was a kind gift from Dr Tanya Paull, and recombinant Plk1 and CK2 protein were purchased from Abcam and New England BioLabs respectively. pCBASceI (Richardson et al., 1998) was obtained from Addgene (gene ID: 26477). Genes encoding AAVS1 targeting zinc-finger domains are synthesised from GeneArt according to the previously described amino acid sequence (Hockemeyer et al., 2009), and cloned into pZFN1 and pZFN2 (Sigma, kind gifts from Prof. Peter Cook) at Acc65I-BamHI sites. To generate pZDonor-AAVS1-GFP, a GFP gene was PCR cloned into pZDonor-AAVS1-Puromycin (Sigma) at BglII-NcoI sites.

Antibodies

Primary antibodies for western blot analyses were obtained from following sources. Anti-BRCA2 (OP95, Calbiochem), anti-Cyclin E (M-20, sc-481, Santa Cruz), anti-Cyclin A (AT10-3, a kind gift from Tim Hunt), anti-FLAG (M2, Sigma-Aldrich), anti-GST-HRP (B-14, Santa Cruz), anti-H2AX (2595, Cell Signaling), anti-γ-H2AX (JBW301, Upstate), anti-Lamin (L1293, Sigma-Aldrich), anti-Nbs1 (34/NBS1, BD Biosciences), anti-Plk1 (A300-251A, Bethyl Laboratories), anti-Plk1 (36-298, Sigma-Aldrich), anti-phospho-T210 Plk1 (558400, BD Biosciences), anti-phospho-S10 Histone H3 (9701, Cell Signalling), anti-PALB2 (A301-246A, Bethyl Laboratories), anti-Rad51 (14B4, Abcam), and anti-Rad51 (FBE2, a kind gift from Stephen West). Phosphopeptide antibodies were raised in rabbit against the KLH-conjugated phosphopeptide (NH2)-CEANADTpSVEEE-(COOH) for S14 phospho-specific antibody,

(NH2)-CEANADpTSVEEE-(COOH) for T13 phospho-specific antibody, or (NH2)-CEANADpTpSVEEES-(COOH) for T13 and S14 double phospho-specific antibody (Biogenes). Antibodies were affinity purified using phosphopeptide columns prepared with SulfoLink Kit (Pierce). After elution with ImmunoPure Gentle Ag/Ab Elution Buffer (Pierce), antibodies were dialysed against TBS and contaminating non-phosphospecific antibody was affinity depleted by passing through a column cross-linked with non-phosphopeptide (NH2)-CEANADTSVEEE-(COOH). The eluted phospho-specific antibodies were then enriched by dialysis against TBS containing 50% glycerol.

Mass Spectrometry Analysis for Identification of Phosphorylation Sites

Polyacrylamide gel slices (1-2 mm) containing the purified proteins were prepared for mass spectrometric analysis using the Janus liquid handling system (PerkinElmer, UK). Briefly, the excised protein gel pieces were placed in a well of a 96-well microtitre plate and destained with 50% v/v acetonitrile and 50 mM ammonium bicarbonate, reduced with 10 mM DTT, and alkylated with 55 mM iodoacetamide. After alkylation, proteins were digested with 6 ng/µL Trypsin (Promega, UK) overnight at 37°C. The resulting peptides were extracted in 2% v/v formic acid, 2% v/v acetonitrile. The digest was analysed by nano-scale capillary LC-MS/MS using a nanoAcquity UPLC (Waters, UK) to deliver a flow of approximately 300 nL/min. A C18 Symmetry 5 µm, 180 µm x 20 mm µ-Precolumn (Waters, UK), trapped the peptides prior to separation on a C18 BEH130 1.7 µm, 75 µm x 100 mm analytical UPLC column (Waters, UK). Peptides were eluted with a gradient of acetonitrile. The analytical column outlet was directly interfaced via a modified nano-flow electrospray ionisation source, with a hybrid linear quadrupole fourier transform mass spectrometer (LTQ Orbitrap XL/ETD, ThermoScientific, San Jose, USA). Data dependent analysis was carried out, using a resolution of 30,000 for the full MS spectrum, followed by eight MS/MS spectra in the linear ion trap. MS spectra were collected with an automatic target gain control of $5x10^5$ and a maximum injection fill time of 100 ms over a m/z range of 300–2000. MS/MS scans were collected using an automatic gain control value of $4x10^4$ and a threshold energy of 35 for collision induced dissociation. LC-MS/MS data were then searched against a protein database (UniProt KB) using the Mascot search engine programme (Matrix Science, UK) (Perkins et al., 1999). Database search parameters were set with a precursor tolerance of 5 ppm and a fragment ion mass tolerance of 0.8 Da. One missed enzyme cleavage was

allowed and variable modifications for oxidized methionine, carbamidomethyl cysteine, pyroglutamic acid, phosphorylated serine, threonine and tyrosine were included. MS/MS data were validated using the Scaffold programme (Proteome Software Inc., USA) (Keller et al., 2002). All data were additionally interrogated manually.

Analysis of Cell Cycle Distribution by Flow Cytometry

U2OS cells were pulse labeled with 10 µM bromodeoxyuridine (BrdU) (Sigma-Aldrich) for 30 min prior to harvesting by trypsinisation. Cells were washed in PBS and fixed with ice cold 70% ethanol for 30 min on ice. Fixed cells were washed in PBS and DNA was denatured with 2 N HCl containing 0.5% Triton X-100 for 30 min at room temperature, followed by neutralisation with 0.1M $Na_2B_4O_7$ at pH 8.5. BrdU incorporation was detected by anti-BrdU (BU1/75 (ICR1), Abcam) at a final concentration of 0.5 µg/ml with 30 min incubation and subsequently by secondary antibody conjugated with 4 µg/ml Alexa Fluor 647 (Invitrogen, A21247) for 30 min in the dark at room temperature. Cells were counter-stained with 20 µg/ml propidium iodide (Sigma-Aldrich) in the presence of 200 µg/ml Ribonuclease A (Sigma-Aldrich) in PBS for 16 hours at 4°C in the dark. Samples were analysed on a Dako CyAn ADP flow cytometer using Summit (4.3) software and gated to distinguish the S phase cell population with BrdU staining and DNA content with PI staining.

Immunofluorescence Microscopy

Cells were seeded at a density of 1 x 10^5 cells in 12-well plates containing coverslips 24 hours after siRNA transfection. After overnight incubation, cells were irradiated with 4 Gy, and coverslips were recovered after 2.5 hours. The cells were pre-extracted for 1 min in pre-extraction buffer (80 mM NaCl, 3 mM $MgCl_2$) and fixed with 4% paraformaldehyde in PBS, followed by permeabilisation with 0.5% Triton X-100 in PBS. After blocking in antibody dilution buffer (1% BSA, 0.2% cold fish skin gelatin and 0.05% Triton X-100 in PBS), primary antibody against Rad51 and γ-H2AX were applied. Following incubation with fluorescently labeled secondary antibody, the coverslips were mounted with ProLong Gold antifade reagent with DAPI (P36935, Invitrogen) on glass slides. Fluorescence images were captured with an Olympus BX60 microscope (Cambridge Research) and Instrumentation model N-MSI-420-20 camera and CRi Nuance Multispectral Imaging System version 2.10.0 software.

Small Interfering RNAs

Following small interfering RNAs were synthesized from Sigma-Aldrich, and a mixture of two siRNA was used to down regulate indicated gene product.

Human siRad51 3'UTR-1	5'-GACUGCCAGGAUAAAGCUU-3'
Human siRad51 3'UTR-2	5'-GUGCUGCAGCCUAAUGAGA-3'
Human siBRCA2-1	5'-CAACAAUUACGAACCAAAC-3'
Human siBRCA2-2	5'-CUGAGCAAGCCUCAGUCAA-3'
Control siRNA-1	5'-GAACAACCUCACAAACUAA-3'
Control siRNA-2	5'-GGCAAACGCAUCACUACGU-3'

Oligonucleotides Used for RAD51 Mutagenesis

Oligonucleotides used for RAD51 mutagenesis were designed using the QuikChange Primer Design tool (Stratagene), and synthesized by Sigma-Aldrich.

S14A	5'-cttgaagcaaatgcagatactgcagtggaagaagaaagctttg-3'
S14A_antisense	5'-caaagctttcttcttccactgcagtatctgcatttgcttcaag-3'
S14E	5'-gcttgaagcaaatgcagatactgaggtggaagaagaaagctttggcc-3'
S14E_antisense	5'-ggccaaagctttcttcttccacctcagtatctgcatttgcttcaagc-3'
S14D	5'-gcttgaagcaaatgcagatactgatgtggaagaagaaagctttggcc-3'
S14D_antisense	5'-ggccaaagctttcttcttccacatcagtatctgcatttgcttcaagc-3'
T13A	5'-gcttgaagcaaatgcagatgcttcagtggaagaagaaag-3'
T13A_antisense	5'-ctttcttcttccactgaagcatctgcatttgcttcaagc-3'
T13AS14A	5'-agatgcagcttgaagcaaatgcagatgctgcagtggaagaagaa-3'
T13AS14A_antisense	5'-ttcttcttccactgcagcatctgcatttgcttcaagctgcatct-3'

SUPPLEMENTAL REFERENCES

Baumann, P., Benson, F.E., Hajibagheri, N., and West, S.C. (1997). Purification of human Rad51 protein by selective spermidine precipitation. Mutat Res *384*, 65-72.

Esashi, F., Galkin, V.E., Yu, X., Egelman, E.H., and West, S.C. (2007). Stabilization of RAD51 nucleoprotein filaments by the C-terminal region of BRCA2. Nat Struct Mol Biol *14*, 468-474.

Keller, A., Nesvizhskii, A.I., Kolker, E., and Aebersold, R. (2002). Empirical statistical model to estimate the accuracy of peptide identifications made by MS/MS and database search. Anal Chem *74*, 5383-5392.

Perkins, D.N., Pappin, D.J.C., Creasy, D.M., and Cottrell, J.S. (1999). Probability-based protein identification by searching sequence databases using mass spectrometry data. Electrophoresis *20*, 3551-3567.

Richardson, C., Moynahan, M.E., and Jasin, M. (1998). Double-strand break repair by interchromosomal recombination: suppression of chromosomal translocations. Genes Dev *12*, 3831-3842.

A Rad51 non-treated: 250/339 (74% coverage)

```
MAMQMQLEANADTSVEEESFGPQPISRLEQCGINANDVKKLEEAGFHTVEAVAYAPKKE
LINIKGISEAKADKILAEAAKLVPMGFTTATEFHQRRSEIIQITTGSKELDKLLQGGIE
TGSITEMFGEFRTGKTQICHTLAVTCQLPIDRGGGEGKAMYIDTEGTFRPERLLAVAER
YGLSGSDVLDNVAYARAFNTDHQTQLLYQASAMMVESRYALLIVDSATALYRTDYSGRG
ELSARQMHLARFLRMLLRLADEFGVAVVITNQVVAQVDGAAMFAADPKKPIGGNIIAHA
STTRLYLRKGRGETRICKIYDSPCLPEAEAMFAINADGVGDAKD
```

B Rad51 Plk1-treated: 289/339 (85% coverage)

```
MAMQMQLEANADTSVEEESFGPQPISRLEQCGINANDVKKLEEAGFHTVEAVAYAPKKE
LINIKGISEAKADKILAEAAKLVPMGFTTATEFHQRRSEIIQITTGSKELDKLLQGGIE
TGSITEMFGEFRTGKTQICHTLAVTCQLPIDRGGGEGKAMYIDTEGTFRPERLLAVAER
YGLSGSDVLDNVAYARAFNTDHQTQLLYQASAMMVESRYALLIVDSATALYRTDYSGRG
ELSARQMHLARFLRMLLRLADEFGVAVVITNQVVAQVDGAAMFAADPKKPIGGNIIAHA
STTRLYLRKGRGETRICKIYDSPCLPEAEAMFAINADGVGDAKD
```

C Rad51 CK2-treated: 284/339 (84% coverage)

```
MAMQMQLEANADTSVEEESFGPQPISRLEQCGINANDVKKLEEAGFHTVEAVAYAPKKE
LINIKGISEAKADKILAEAAKLVPMGFTTATEFHQRRSEIIQITTGSKELDKLLQGGIE
TGSITEMFGEFRTGKTQICHTLAVTCQLPIDRGGGEGKAMYIDTEGTFRPERLLAVAER
YGLSGSDVLDNVAYARAFNTDHQTQLLYQASAMMVESRYALLIVDSATALYRTDYSGRG
ELSARQMHLARFLRMLLRLADEFGVAVVITNQVVAQVDGAAMFAADPKKPIGGNIIAHA
STTRLYLRKGRGETRICKIYDSPCLPEAEAMFAINADGVGDAKD
```

Figure S1. Mass Spectrometry Analyses, Related to Figures 1 and 3

(A–C) *In vitro* phosphorylated Rad51 was analysed by mass spectrometry as described in Experimental Procedures. Detected peptides cover 74%, 85%, and 84% of full length Rad51 sample treated with no kinase (A), Plk1 (B), or CK2 (C) respectively, which is highlighted in red. The identified phosphorylated residues are highlighted in yellow.

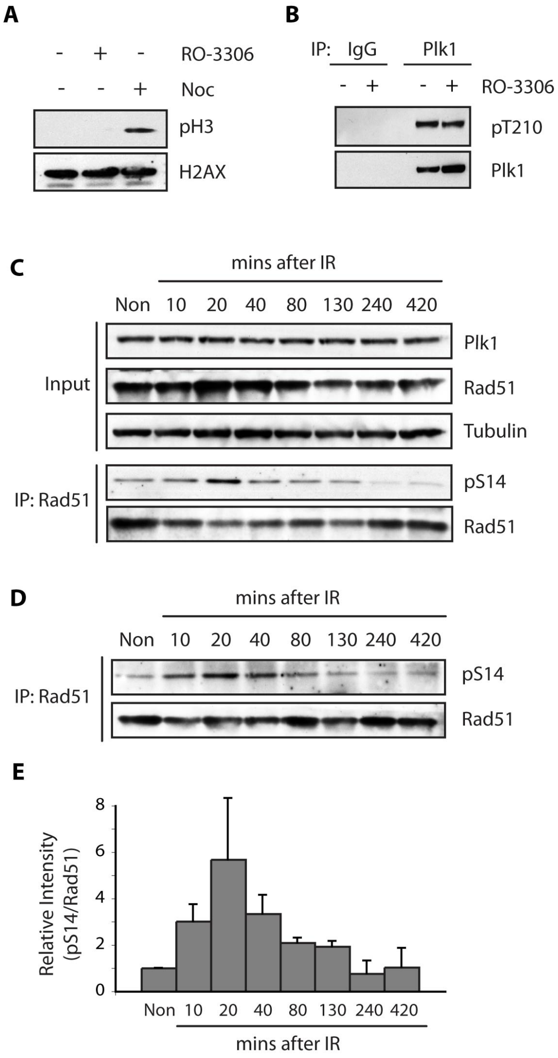
A
- + -
RO-3306
- - +
Noc
pH3
H2AX
B
IP: IgG Plk1
- + - +
RO-3306
pT210
Plk1
C
mins after IR
Non 10 20 40 80 130 240 420
Input
Plk1
Rad51
Tubulin
IP: Rad51
pS14
Rad51
D
mins after IR
Non 10 20 40 80 130 240 420
IP: Rad51
pS14
Rad51
E
Relative Intensity (pS14/Rad51)
0
2
4
6
8
Non 10 20 40 80 130 240 420
mins after IR

Figure S2. Evaluation of Cells Treated with RO-3306, and Rad51 S14 Phosphorylation after Irradiation, Related to Figure 4

(A) HeLa cells were treated with RO-3306 or nocodazole (Noc), and whole cell extract was blotted with phospho-S10 Histone H3 antibody. The same membrane was blotted using pan-H2AX antibody as a loading control. Unlike in nocodazole treated cells (lane 3), phosphorylated histone H3, a mitotic marker, was not detected in RO-3306 treated cells (lane 2) in agreement that RO-3306 treatment arrests cells in G2 phase.

(B) The active form of Plk1 with T210 phosphorylation (pT210) was detected in RO-3306 treated cells, consistent with previous reports that Plk1 activity is present before the onset of mitosis.

(C) HeLa cells were treated with ionizing radiation (4 Gy), and Rad51 phosphorylation was analysed with the pS14 antibody. The total amount of Plk1 did not change after DNA damage, whilst a transient increase of S14 phosphorylation was detected.

(D) Repeat analysis of IR-induced p14 phosphorylation as (C).

(E) Relative intensity of S14 phosphorylated Rad51 against total Rad51 in (C) and (D) are quantified, and their average are shown. Error bars: one s.d. (n=2).

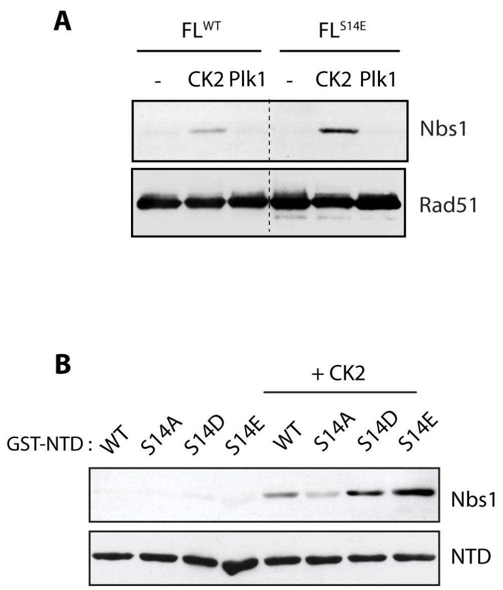

Figure S3. Interaction between Rad51 and Nbs1, Related to Figure 5

(A) Recombinant wild type full length Rad51 (FL^{WT}) or its variant harbouring S14E substitution (FL^{S14E}) was phosphorylated *in vitro* with CK2 or Plk1, and Nbs1 interaction was tested by far-western blotting using recombinant Nbs1 as described in Figure 5A.

(B) Similarly, Rad51 NTD variants with S14 substitution were phosphorylated with CK2, and its interaction with Nbs1 was detected by far-western blotting. Consistent with the altered CK2-mediated phosphorylation of Rad51 S14 variants shown in Figure 3D, increased Nbs1 interactions with CK2 phosphorylated S14D or S14E NTD, but reduced interaction with S14A NTD, were detected.

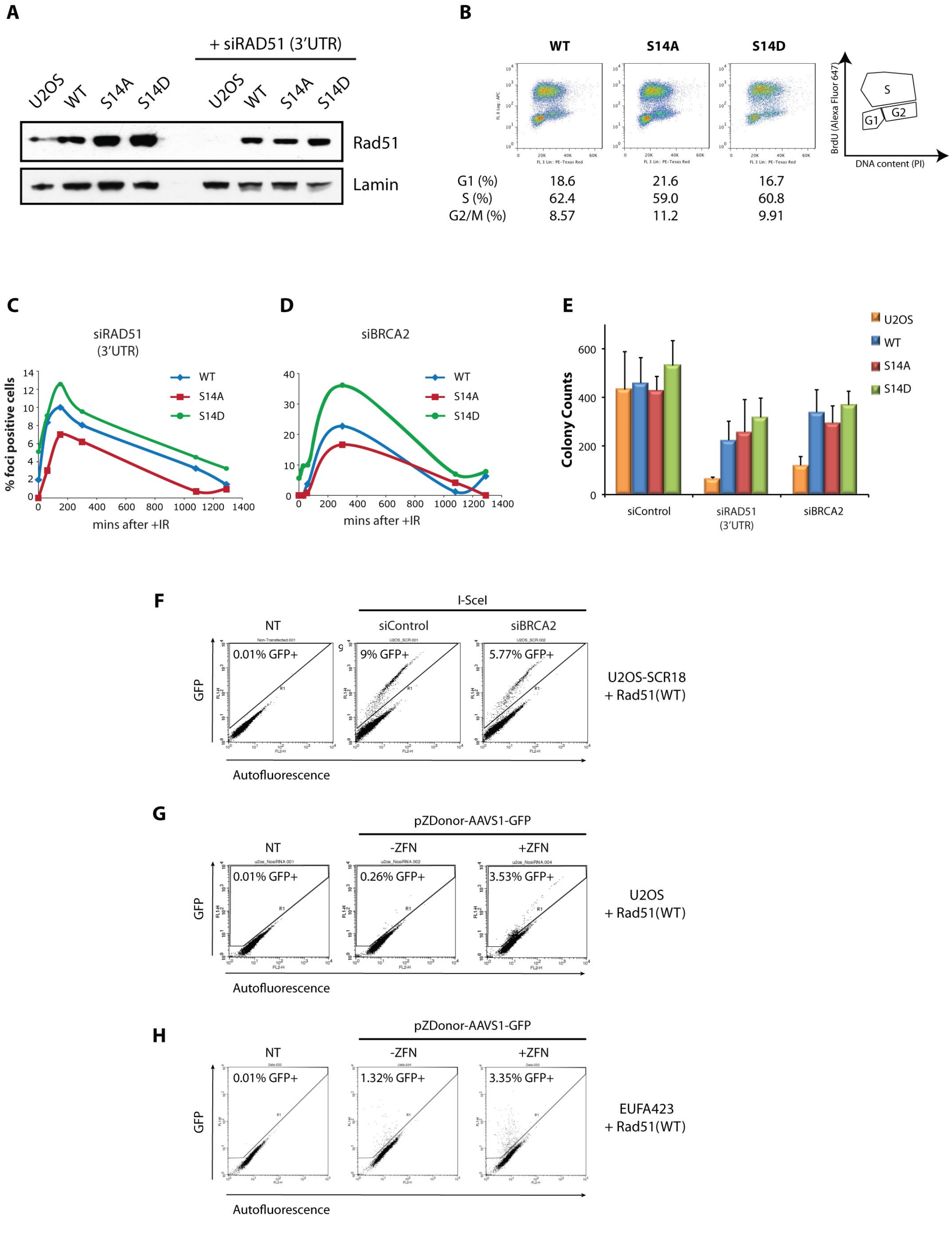
A
+ siRAD51 (3'UTR)
U2OS
WT
S14A
S14D
U2OS
WT
S14A
S14D
Rad51
Lamin
B
WT
S14A
S14D
BrdU (Alexa Fluor 647)
DNA content (PI)
S
G1
G2
G1 (%) 18.6 21.6 16.7
S (%) 62.4 59.0 60.8
G2/M (%) 8.57 11.2 9.91
C
siRAD51
(3'UTR)
% foci positive cells
mins after +IR
WT
S14A
S14D
D
siBRCA2
mins after +IR
WT
S14A
S14D
E
Colony Counts
U2OS
WT
S14A
S14D
siControl
siRAD51
(3'UTR)
siBRCA2
F
I-SceI
NT
siControl
siBRCA2
0.01% GFP+
9% GFP+
5.77% GFP+
GFP
Autofluorescence
U2OS-SCR18
+ Rad51(WT)
G
pZDonor-AAVS1-GFP
NT
-ZFN
+ZFN
0.01% GFP+
0.26% GFP+
3.53% GFP+
GFP
Autofluorescence
U2OS
+ Rad51(WT)
H
pZDonor-AAVS1-GFP
NT
-ZFN
+ZFN
0.01% GFP+
1.32% GFP+
3.35% GFP+
GFP
Autofluorescence
EUFA423
+ Rad51(WT)

Figure S4. Phenotypes of Cells Expressing Rad51 Variants, Related to Figure 6

(A) Stable expression of S14 Rad51 variants in U2OS cells was confirmed after down-regulating endogenous Rad51 with siRNA targeting the 3' UTR. Lamin was used as a loading control.

(B) Cell cycle profiles of S14 Rad51 variant expressing cells. Cells were pulse-labeled with 5-bromodeoxyuridine to detect cells actively replicating DNA, and DNA contents were detected with PI staining using flow cytometry.

(C and D) Cells treated with siRad51 (C) or siBRCA2 (D) were irradiated at 4 Gy, and cells containing more than twenty Rad51 foci at indicated time after IR was counted.

(E) Five hundred cells with down-regulated endogenous Rad51 or BRCA2 by siRNA were seeded, and the resulting colonies were counted after two weeks. There was no significant difference in clonogenic survival among cells expressing different Rad51 variant. Error bars s.d. (n=3).

(F) An example of HR assay depicted in Figure 6H, showing a representative flow cytometric profile of U2OS-SCR18 cells exogenously expressing wild-type Rad51.

(G and H) Examples of DSB-mediated gene targeting assay depicted in Figure 6J, showing a representative flow cytometric profile of U2OS cells exogenously expressing wild-type Rad51 (G) or EUFA423 cells exogenously expressing wild-type Rad51 (H).
